# Atezolizumab and Bevacizumab Combination Therapy in the Treatment of Advanced Hepatocellular Cancer

**DOI:** 10.3390/cancers16010197

**Published:** 2023-12-30

**Authors:** Ignacio Ventura, Lorena Sanchiz, María Ester Legidos-García, María Teresa Murillo-Llorente, Marcelino Pérez-Bermejo

**Affiliations:** 1 Molecular and Mitochondrial Medicine Research Group, School of Medicine and Health Sciences, Catholic University of Valencia San Vicente Mártir, C/Quevedo nº 2, 46001 Valencia, Spain; ignacio.ventura@ucv.es; 2 Translational Research Center San Alberto Magno CITSAM, Catholic University of Valencia San Vicente Mártir, C/Quevedo nº 2, 46001 Valencia, Spain; 3 School of Medicine and Health Sciences, Catholic University of Valencia San Vicente Mártir, C/Quevedo nº 2, 46001 Valencia, Spain; lorenasanchiz@mail.ucv.es; 4 SONEV Research Group, Faculty of Medicine and Health Sciences, Catholic University of Valencia San Vicente Mártir, C/Quevedo nº 2, 46001 Valencia, Spain; ester.legidos@ucv.es (M.E.L.-G.); mt.murillo@ucv.es (M.T.M.-L.)

**Keywords:** liver cancer, liver neoplasms, hepatocellular cancer, hepatocellular carcinoma, Sorafenib, Atezolizumab, Bevacizumab

## Abstract

**Simple Summary:**

This research aims to investigate the effectiveness of combining Atezolizumab and Bevacizumab as a potential treatment for advanced liver cancer (hepatocellular carcinoma or HCC). The authors reviewed multiple studies and clinical trials to assess the impact of this combination therapy on patient survival and disease progression. While the results show promising benefits in terms of increased overall survival for HCC patients, the treatment also carries significant side effects. Additionally, there is a lack of consensus on specific biomarkers to predict treatment outcomes. This study highlights the need for personalized treatment approaches and further research to optimize the management of this deadly disease.

**Abstract:**

Liver cancer, particularly hepatocellular carcinoma, is a global concern. This study focuses on the evaluation of Atezolizumab and Bevacizumab combination therapy as a promising alternative in the treatment of advanced hepatocellular carcinoma. The objectives of this systematic review include evaluating the efficacy of Atezolizumab and Bevacizumab combination therapy compared to conventional therapies with Sorafenib and other conventional therapies, analyzing the associated adverse effects, and exploring prognostic factors in the setting of advanced hepatocellular carcinoma. A systematic literature review was carried out using the PubMed and Web of Science databases. Fifteen related articles were included and evaluated according to their level of evidence and recommendation. Results: The combination therapy of Atezolizumab and Bevacizumab, along with Sorafenib, showed positive results in the treatment of patients with advanced hepatocellular carcinoma. Significant adverse effects were identified, such as gastrointestinal bleeding, arterial hypertension, and proteinuria, which require careful attention. In addition, prognostic factors, such as transforming growth factor beta (TGF-β), alpha-fetoprotein (AFP), and vascular invasion, were highlighted as key indicators of hepatocellular carcinoma progression. Conclusions: The combination of Atezolizumab and Bevacizumab is shown to be effective in the treatment of advanced hepatocellular carcinoma, although it is essential to take into consideration the associated adverse effects. The prognostic factors identified may provide valuable information for the clinical management of this disease. This study provides a comprehensive overview of a promising emerging therapy for liver cancer.

## 1. Introduction

Hepatocellular carcinoma (HCC) is the most prevalent primary neoplasm affecting the liver, emerging as a preeminent cause of mortality, particularly in patients with liver cirrhosis. The incidence of this cancer varies according to geographic location and risk factors, with a higher frequency in males [1,2]. Globally, HCC ranks sixth in terms of prevalence among neoplasms and is the third-leading cause of death from oncologic diseases. The age of HCC onset and survival rates are subject to regional variations. In nations such as Taiwan and Japan, where it tends to be diagnosed later in life, there is longer survival due to early detection. In contrast, in areas such as Korea, China, North America, and Europe, most cases are identified in intermediate or advanced stages of the disease [3].

In Spain, in 2019, a total of 6499 cases of liver cancer were reported [2,4,5], being more prevalent in men. Risk factors include liver inflammation, which can lead to cirrhosis, as well as the presence of diabetes, obesity, dyslipidemia, and excessive alcohol consumption. HCC is associated with viral infections, such as hepatitis B virus (HBV) and hepatitis C virus (HCV), in addition to alcoholic cirrhosis and nonalcoholic fatty liver disease (NAFLD), with diabetes and obesity acting as contributing factors [3,6]. Additional risk factors include male gender, advanced age (greater than 65 years), the presence of cirrhosis, chronic alcohol abuse, genotype 3, diabetes, metabolic syndrome, low albumin/platelet levels, and elevated alpha-fetoprotein (AFP) levels. In contrast, protective factors include HBV vaccination, antiviral therapy in the setting of chronic hepatitis, abstention from coffee and alcohol consumption in chronic liver conditions, and the adoption of a healthy lifestyle. In the context of chronic liver conditions and the risk of HCC, adopting a healthy lifestyle involves practices such as maintaining a balanced diet, engaging in regular physical activity, abstaining from excessive alcohol consumption, and avoiding harmful habits like smoking. These lifestyle choices are considered protective factors that may contribute to reducing the likelihood of developing liver cancer, complementing medical interventions and antiviral therapies for chronic liver diseases [5,7,8].

Primary prevention focuses on the reduction of HCC in low-income countries through measures to prevent hepatitis B virus (HBV) transmission, the sterilization of surgical instruments, and the quality control of blood products. Secondary prevention focuses on early detection through ultrasound scans performed every 6 months in patients considered to be at high risk, including those with cirrhosis and chronic hepatitis B. Early diagnosis is of critical importance and is based on both radiological screening techniques, such as abdominal ultrasound, and serological screening, including measurement of AFP. The combination of both strategies proves to be more effective [4,5].

The diagnosis is based on specific radiological features evaluated by computed tomography (CT) and magnetic resonance imaging (MRI) [9]. In patients with cirrhosis, the diagnosis is considered without resorting to a liver biopsy. For nodules less than 1 cm in diameter, follow-up every 3–4 months is recommended. For nodules 1–3 cm, the diagnosis can be established without pathological confirmation in patients with cirrhosis or chronic hepatitis B. However, for other cases, follow-up every 3–4 months and further pathological confirmation are recommended [5,10,11,12,13].

The therapeutic approach to HCC encompasses various modalities, including surgical resection, ablation, radiotherapy, immunotherapy, liver transplantation, chemotherapy, and targeted therapy. However, recurrence is a common event, particularly after resection or ablation. Liver resection is characterized by its high cure rate, although recurrence persists as a relevant complication. As for liver transplantation, it represents a definitive option if there are no metastases present. Local ablation, through radiofrequency, is effective, especially in the treatment of small tumors.

In the context of systemic treatment, Sorafenib and Lenvatinib are agents used in the first line of therapy, while Regorafenib and other agents are reserved for the second line. The choice of treatment is based on patient- and tumor-specific characteristics [5,14,15,16]. Transforming growth factor beta (TGF-β) plays a critical role in biological processes and the progression of HCC. Alterations in it signaling pathway can drive tumor progression. Mutations in SMAD and TGF-β receptor genes have been identified in several types of cancer, supporting their suppressive role. Their influence on immune responses varies depending on the context. In summary, HCC manifests as a complex disease that involves multiple risk factors and shows remarkable geographic variations in its incidence and survival [17,18,19,20].

**Figure 1:**
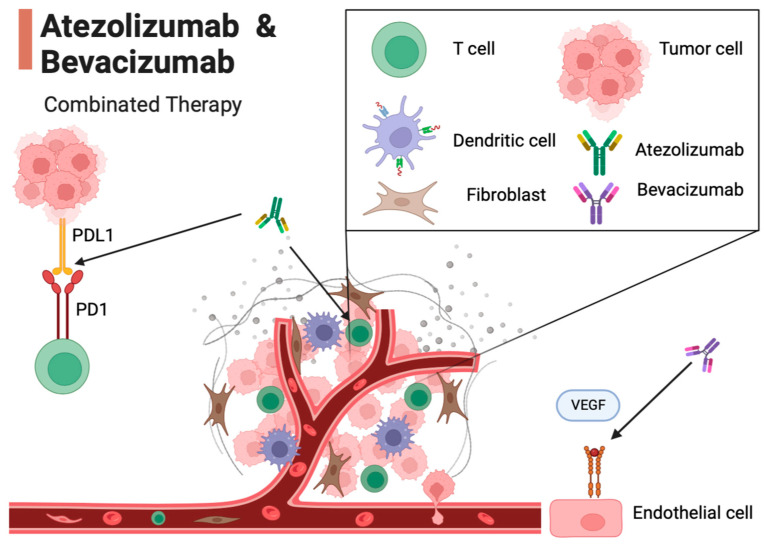
Mechanism of action of Atezolizumab and Bevacizumab in the tumor microenvironment. In this figure, key interactions in the tumor microenvironment are illustrated. T cells are activated by Atezolizumab, an immune checkpoint inhibitor. Atezolizumab inhibits the interaction between the PD-1 receptor on T cells and the PD-L1 on tumor cells, enabling T cells to target tumor cells. Endothelial cells are influenced by Bevacizumab, a monoclonal antibody that targets VEGF. Bevacizumab hinders the growth of new blood vessels (angiogenesis) by blocking VEGF, preventing tumor cells from receiving the nutrients and oxygen required for growth. This combination of mechanisms of action demonstrates a synergistic effect in the battle against cancer, potentially extending the survival of patients with advanced HCC.

The combination of Atezolizumab and Bevacizumab represents a therapeutic approach for advanced hepatocellular carcinoma (HCC), operating through distinct mechanisms: Atezolizumab serves as an immune checkpoint inhibitor, targeting proteins known as immune checkpoints, which regulate the immune response. Atezolizumab exerts its action by antagonizing the PD-1 receptor expressed on T cells and the PD-L1 protein found on tumor cells. This antagonism results in the disruption of an inhibitory interaction, enabling T cells to mount a more robust assault against tumor cells. Conversely, Bevacizumab is a monoclonal antibody that selectively targets the vascular endothelial growth factor (VEGF), a pivotal protein orchestrating angiogenesis—the formation of new blood vessels. By obstructing VEGF, Bevacizumab effectively curtails the growth of blood vessels that nourish tumors. The confluence of these two distinct mechanisms engenders a synergistic therapeutic effect that surpasses the efficacy of each drug in isolation. More specifically, Atezolizumab enhances the capability of the immune system’s T cells to identify and eliminate tumor cells. Bevacizumab, on the other hand, disrupts the tumor cells’ supply of vital nutrients and oxygen, impeding their capacity for growth [17,18,19,20,21,22,23] (Figure 1).

The main objective of this systematic review will therefore be to evaluate the influence of the use of Atezolizumab in combination with Bevacizumab compared to Sorafenib treatment in patients with hepatocellular carcinoma, as well as to analyze its adverse effects.

## 2. Search Methodology

This systematic review was conducted in accordance with the criteria set out in the Preferred Reporting Items for Systematic Reviews and Meta-Analysis (PRISMA) guidelines [24,25]. The protocol has not been registered. The literature search was performed in PubMed, Cochrane, and Web of Science. The search strategy was carried out by combining the following MeSH terms using Boolean operators: “Transforming Growth Factor β”, “liver cancer”, “liver neoplasms”, “hepatocellular cancer”, hepatocellular carcinoma”, “Sorafenib”, “Atezolizumab”, and “Bevacizumab”. The search equation was ((((((((TGF β) OR (Transforming Growth Factor β)) AND (liver cancer)) OR (liver neoplasms)) OR (hepatocellular cancer)) OR (hepatocellular carcinoma)) AND (Sorafenib)) AND (Atezolizumab)) AND (Bevacizumab).

**Figure 2:**
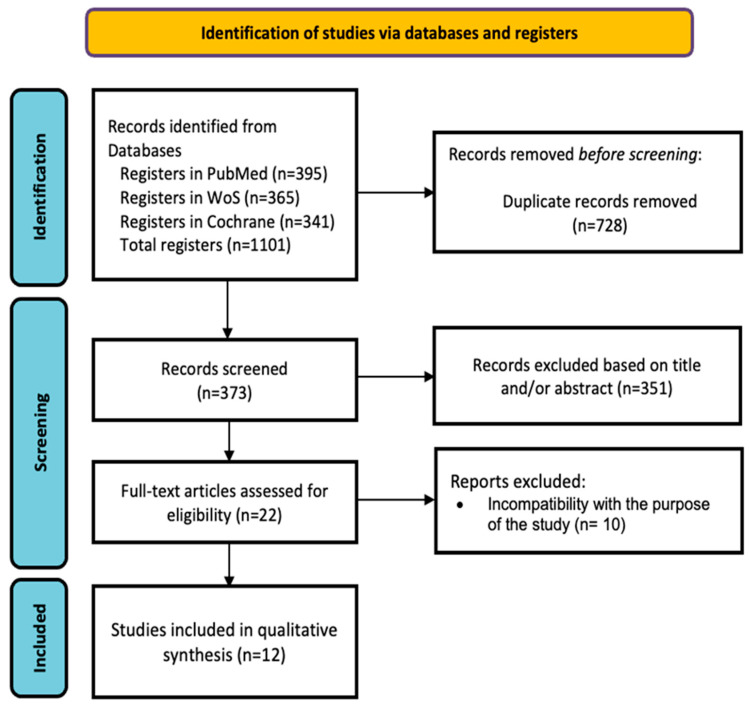
PRISMA flowchart of the study selection process.

The initial search resulted in a total of 1101 articles. Human studies published in the last 5 years in full text in English evaluating the combination of Atezolizumab and Bevacizumab in the treatment of HCC and its adverse effects were included. The flow diagram in Figure 2 describes the screening and selection process. All studies were based on randomized controlled trials, and the quality of these articles was high, as assessed by the Joanna Briggs Institute (JBI) checklist for randomized clinical trials [26]. The bibliographic search continued with narrowed results after establishing the selection criteria for the articles of interest. Inclusion criteria were studies conducted with human subjects, published in the last 5 years in the English language, with full-text availability and with high scientific evidence. Studies conducted exclusively with animals or studies on TGF-β based solely on cancer at the general level, with no direct relation to the combination of Atezolizumab + Bevacizumab in the treatment of HCC, were considered as exclusion criteria [17].

## 3. Results

As shown in Figure 2, 22 articles were selected for full-text review, of which 12 studies met the inclusion criteria. Table 1 shows the main characteristics of each study, and Table 2 shows the quality assessment of the studies.

This systematic analysis summarizes key findings from various studies evaluating the efficacy and safety of the combination of Atezolizumab and Bevacizumab in the treatment of advanced HCC. The studies reveal that this combination demonstrates a positive response in patients with advanced HCC, with higher overall survival and progression-free survival rates compared to the previous standard, Sorafenib [27,28,36]. Table 3 presents the overall survival (OS) data reported in the analyzed studies of treatment for advanced hepatocellular carcinoma. The data indicate that treatment with Atezolizumab and Bevacizumab provides a significant increase in survival compared to other treatments such as Atezolizumab alone, Sorafenib, Nivolimab, Lenvatinib, Linifanib, or Sunitinib.

Furthermore, the importance of considering prognostic factors, such as PD-L1 expression and biomarkers like VEGF receptor 2 [34,35], for more precise patient selection is emphasized. While the efficacy of Atezolizumab and Bevacizumab is highlighted, the urgency to develop more robust biomarkers for further personalized HCC treatment is underscored [29,30,31,32]. The studies also address treatment sequencing, highlighting that this combination might be especially promising in patients with virally induced HCC [35,36,37]. Despite its benefits, the side effects, although manageable, suggest the need for close monitoring during therapy. Overall, these studies emphasize the need for a personalized approach in managing HCC [28,30,35], considering the diversity of patient subgroups and the lack of robust biomarkers in therapeutic decision-making [28,37,38]. The combination of Atezolizumab and Bevacizumab emerges as a promising option, but careful management of its adverse effects and the precise identification of suitable patients are essential to maximize the benefits and minimize the risks [37].

## 4. Discussion

In the present systematic review, we address key aspects related to hepatocellular carcinoma, a liver cancer of great relevance in the field of oncology. HCC manifests as the predominant type of primary hepatic neoplasm and is frequently associated with high mortality rates, especially in patients with a history of liver cirrhosis. Currently, HCC therapeutics include a variety of first- and second-line drugs.

This systematic review focuses on comprehensively investigating the results of representative studies evaluating the combination therapy of the drugs Atezolizumab and Bevacizumab as an alternative to conventional systemic treatment in patients with HCC in the adult population. In addition, the adverse effects associated with this therapy are addressed, and possible predictive biomarkers of disease progression are explored.

In the phase III IMBrave150 study, led by Cheng et al. [34], the combination therapy of Atezolizumab and Bevacizumab was compared to the conventional treatment, Sorafenib, in terms of overall survival and progression-free survival. A survival benefit was seen in patients treated with the combination of Atezolizumab and Bevacizumab, supporting the efficacy of this therapeutic approach. These results were corroborated in the work of Zhang et al. [21] regarding overall survival. In addition, Fulgenzi et al. [36] noted a significantly higher rate of a radiologically measurable response in patients treated with this combination therapy.

Consistent findings were observed in the work of Sonbol et al. [31] in comparing Sorafenib with the combination of Atezolizumab and Bevacizumab and in a comparative study by Lee et al. [29] in comparing Atezolizumab and Bevacizumab with Atezolizumab monotherapy. Finn et al. [27] conducted a global open-label phase III trial that also supported the superiority of combination therapy in terms of overall survival at 12 months, particularly in previously untreated patients. However, Pinter et al. [23] warned about the contraindication of this combination therapy in patients who had previously received an organ transplant.

Additionally, in studies involving the drug Lenvatinib, such as the analyses by Fulgenzi et al. [33] and Piñero et al. [28], superior survival was observed in patients treated with the Atezolizumab and Bevacizumab combination, followed by the Lenvatinib group. Han et al. [32] supported these results, although they noted a higher incidence of treatment interruptions due to adverse effects in combination therapy.

On the other hand, Rimini et al. [37] put forward a different perspective by suggesting a longer overall survival in patients with advanced HCC treated with Lenvatinib compared to the other two alternatives. These authors disagreed with previous studies, such as those mentioned above, which found no significant differences between patients treated with Atezolizumab and Bevacizumab and Sorafenib [34,38].

Concerning the adverse effects of Atezolizumab combined with Bevacizumab therapy for HCC, Cheng et al. [34] reported grade 3 adverse effects in 43% of patients, including intestinal bleeding and gastric ulcers. In this context, “grade 3” indicates the severity of adverse effects, with a higher grade signifying more significant complications. Specifically, a grade 3 adverse effect denotes substantial severity, as reported in 43% of patients, including instances of intestinal bleeding and gastric ulcers, according to Cheng et al. [34]. Finn et al. [27] also noted serious side effects in 38% of patients.

Han et al. [32] and Zhang et al. [21] reported the occurrence of adverse effects, although they did not provide specific details. Pinter et al. [35], in addition to gastrointestinal bleeding, reported cases of arterial hypertension and proteinuria, adverse effects that were also observed in the multicenter study by Lee et al. [29] and in the study of patients with HCC of viral etiology by Fulgenzi et al. [36].

As HCC is among the deadliest cancers worldwide, the identification of predictive biomarkers is of utmost importance. Pinter et al. [35] suggested TGF-β signaling as a biomarker, proposing that less-altered levels of this cytokine are associated with a better prognosis. However, these authors disagreed regarding PD-L1 expression and tumor burden as predictive factors. Zhang et al. [21] also mentioned TGF-β, correlating it with decreased survival, although they differed regarding PD-L1 expression, correlating it with accelerated HCC progression.

Cheng et al. [34] associated elevated VEGF levels with a greater benefit from therapy, a finding that was also proposed by Pinter et al. [35]. Piñero et al. [28] suggested the absence of extrahepatic pathology and viral etiology of hepatitis C as predictors of longer survival to treatment, whereas the presence of elevated AFP levels and vascular invasion at the macroscopic level were considered poor prognostic factors. On the other hand, Da Fonseca et al. [31] proposed age, cirrhosis, and portal hypertension as influential factors in the prognosis of HCC treatment.

In summary, this review addresses various aspects related to the treatment of HCC using the combination of the drugs Atezolizumab and Bevacizumab. It highlights the significant benefits in terms of survival and response rates observed in several studies, supporting the efficacy of this combination therapy in advanced HCC [37]. In addition, associated adverse effects and potential predictive biomarkers that may influence the prognosis and response to HCC treatment have been explored [38]. These findings offer valuable information for clinical decision-making in the management of this highly lethal disease.

### Study Limitations

Despite efforts to comprehensively evaluate the effectiveness and adverse effects of the combination of the drugs Atezolizumab and Bevacizumab as an alternative to conventional systemic therapy in the treatment of HCC, it is essential to recognize certain limitations that affect the interpretation of the results and the generalizability of the conclusions.

First, we must point out that most of the studies included in this review were based on data from clinical trials and observational studies, which could introduce selection bias and potentially limit the representativeness of the HCC patient population. In addition, the variability in the design methodology of the selected studies, as well as differences in the patient populations, could influence the quality of the evidence and the ability to synthesize the results in a homogeneous manner.

Second, most of the included clinical trials and observational studies had short-term follow-ups in relation to the chronic and evolving nature of HCC. This limitation could influence the ability to assess long-term survival, duration of the treatment response, and the potential occurrence of late adverse effects fully and accurately. In addition, it is necessary to recognize that the assessment of adverse effects is based on the information available in the included studies, and the reporting of these events may be subject to reporting biases. Therefore, it is essential to consider the possibility of underestimation or overestimation of the frequency and intensity of adverse effects associated with Atezolizumab and Bevacizumab therapy [32].

Finally, although a comprehensive effort has been made to identify and analyze the adverse effects associated with this combination therapy and to investigate the prognostic factors that influence the treatment of advanced HCC, the heterogeneity of the data and the lack of standardization in the presentation of the results could limit the ability to perform a robust quantitative analysis.

## 5. Conclusions

The study results suggest a high overall survival rate and superior benefits in patients with advanced HCC who are treated with the combination of Atezolizumab and Bevacizumab compared to conventional therapies. These findings support the efficacy of this combination therapy as a promising alternative in the treatment of this disease. Notwithstanding the successful results observed in terms of survival, it is essential to highlight that several authors reported the appearance of serious adverse effects in a significant percentage of the patients treated with Atezolizumab and Bevacizumab. These adverse effects included gastrointestinal bleeding, arterial hypertension, proteinuria, and gastric ulcers, which pose challenges in terms of the tolerability and safety of this combination therapy.

There is a lack of consensus and clarity regarding the predictive biomarkers of HCC. However, some authors have suggested that elevated levels of TGF-β, AFP, and vascular invasion are associated with an unfavorable prognosis for survival. On the other hand, it has been proposed that elevated VEGF levels, the absence of extrahepatic pathology, and the viral etiology of hepatitis C may predict longer survival in response to treatment. In addition, age, the presence of cirrhosis, and hypertension are mentioned as factors that may influence the prognosis of HCC treatment.

Despite the observed efficacy in terms of survival in patients with advanced HCC treated with Atezolizumab and Bevacizumab, the occurrence of serious adverse effects and the lack of definitive biomarkers must be considered. These results emphasize the need for an individualized approach in clinical decision-making and highlight the importance of future research to address the challenges and optimize the management of this disease.

## Figures and Tables

**Table 1:** Main characteristics of each study analyzed.

Ref.	Year	n	Objective	Conclusions
[27]	2020	165	This article examines the efficacy of the combination of Atezolizumab and Bevacizumab in patients with unresectable HCC. The results suggest that this therapy could be promising, as it showed a positive response rate in patients with advanced HCC. However, associated serious adverse effects, such as gastrointestinal bleeding, arterial hypertension, and proteinuria, which require medical attention, were also highlighted. In addition, prognostic factors, such as TGF-β, AFP, and vascular invasion, were exploited as indicators of HCC progression.	The results showed that, at 12 months, the overall survival rate was 54.6% in the group treated with Sorafenib and 67.2% in the group treated with Atezolizumab and Bevacizumab, suggesting a superior benefit in the second group. However, serious side effects were reported in 38% of patients receiving combination therapy. This highlights the potential efficacy of Atezolizumab and Bevacizumab in the treatment of HCC but also underscores the need to manage the side effects associated with this therapy.
[28]	2020	6125	This article focuses on the sequencing of systemic treatment for HCC and discusses second-line treatment options for patients who have progressed after first-line therapy. Various second-line therapies, including second-line competitors, are discussed, and the importance of the appropriate selection of therapy according to individual patient needs is addressed. The article provides valuable information on the options available for the treatment of advanced stage HCC and highlights the need for personalized attention in therapeutic decision-making.	It is concluded that the combination of the drugs Atezolizumab and Bevacizumab represents an innovative and promising treatment, followed by Lenvatinib, which shows a median survival of 13.6 months in patients with advanced HCC. In addition, predictors of longer survival in patients treated with Sorafenib are identified, including the absence of extrahepatic pathology, the presence of hepatitis C, elevated AFP levels (>200 ng/mL), and the absence of vascular invasion at the macroscopic level. These findings provide useful information for patient stratification and therapeutic decision-making in advanced HCC.
[29]	2020	223	A multicenter phase 1b study evaluated the use of Atezolizumab, alone or in combination with Bevacizumab, in patients with unresectable HCC. The objective was to investigate the safety and efficacy of these therapies. The study was conducted in an open-label manner and revealed valuable information on the potential of these treatment options in patients with unresectable HCC.	Two groups of patients with unresectable HCC were evaluated. Group A (104 patients) received treatment with Atezolizumab and Bevacizumab, and Group F (119 patients) was treated with Atezolizumab alone. Group A had a mean follow-up of 12.4 months, and 36% of patients had a satisfactory response to treatment. In Group F, with a mean follow-up of 6.6 months, adverse effects such as hypertension were observed in 13% and proteinuria in 7% of the patients who received the combined therapy, compared to hypertension in 5% of the patients treated with Atezolizumab alone. These results highlight the efficacy and adverse effects associated with these therapies in patients with unresectable HCC.
[30]	2020	8943	This article discusses systemic therapy options and sequencing in the treatment of advanced HCC. It reviews the different therapies available for advanced HCC and discusses how to select the appropriate sequence of treatments. Effectiveness, tolerability, and clinical considerations in choosing between therapies are discussed, and the importance of personalized care for each patient with advanced HCC is highlighted.	It was shown that patients with unresectable HCC treated with the combination of Atezolizumab and Bevacizumab (a total of 6290 patients) had better outcomes compared to those treated with first-line drugs such as Sorafenib (a total of 2653 patients). The findings suggest that the combination of Atezolizumab and Bevacizumab may be a more effective treatment option for patients with unresectable HCC compared to conventional first-line therapies.
[31]	2021	2198	This study analyzes the eligibility of underrepresented subgroups in clinical trials for advanced hepatocellular carcinoma. They assess whether current clinical practice is adequate for these subgroups and whether clinical trials sufficiently include them. The objective is to determine whether disparities exist in the representation of these subgroups in clinical research and whether greater attention to the inclusion of these patients in trials is required to ensure that treatments are equitable and effective for all groups of patients with advanced hepatocellular carcinoma.	It is suggested that the combination of Atezolizumab and Bevacizumab may have a smaller benefit than Sorafenib in cases of HCC of nonviral etiology, with a HR of 0.91 and a 95% CI of 0.51–1.60. In contrast, for hepatitis B virus (HBV)-related cases, the HR was 0.51 (95% CI 0.32–0.81), and for hepatitis C virus (HCV)-related cases, the HR was 0.43 (95% CI 0.22–0.87). Furthermore, the study suggests that factors such as age, cirrhosis, hepatic decompensation, and portal hypertension may influence the prognosis of HCC treatment. These findings highlight the importance of considering the etiology of HCC and other clinical factors when selecting the appropriate therapy for these patients.
[32]	2021	10,256	This meta-analysis focused on the selection of first-line systemic therapies for advanced cancer. The investigators analyzed multiple clinical trials to determine which of the available therapies offer the best results in terms of efficacy and safety. This analysis allows for a comprehensive comparison between the different therapeutic options available and may provide valuable recommendations for the choice of first-line therapy in patients with advanced HCC.	Greater benefits in terms of overall survival were observed with the combination of Atezolizumab and Bevacizumab compared to Lenvatinib and Sorafenib. The group treated with Atezolizumab and Bevacizumab was found to have a significantly lower death rate compared to the other groups. However, the Atezolizumab- and Bevacizumab-treated group experienced a higher incidence of treatment discontinuations due to adverse effects, which included bleeding and other events. These findings suggest that the combination of Atezolizumab and Bevacizumab may offer benefits in terms of survival but with a higher risk of side effects compared to other therapies.
[33]	2021	7881	This article addresses the topic of immunotherapy in HCC. Immunotherapy is a therapeutic approach used to treat HCC, a type of liver cancer. Immunotherapy involves stimulating the patient’s immune system to fight cancer cells. The text provides information on the status of immunotherapy in the treatment of HCC, including different immunological approaches and therapies used. Recent advances in the understanding of how the immune system interacts with liver cancer cells and how more effective treatments can be developed are also discussed.	A clear superiority was found in terms of increased survival compared to Sorafenib. This analysis suggests that treatment alternatives, such as the drug Lenvatinib, may be more effective in this patient population. However, the study also highlighted the lack of existing predictive biomarkers to accurately target HCC therapy. This underscores the need for research and development of biomarkers that can help to personalize the treatment of this disease.
[34]	2022	501	This phase III study provides updated data on the efficacy and safety of the combination of Atezolizumab and Bevacizumab compared to Sorafenib for the treatment of unresectable hepatocellular carcinoma. The results indicate that this combination continues to show significant benefits in terms of efficacy compared to Sorafenib and remains a promising option for the treatment of this disease. In addition, it is noted that the safety of this therapy has also been supported by updated data from the IMBrave150 study.	The combination of Atezolizumab and Bevacizumab was confirmed to provide longer overall survival (19.2 months vs. 13.4 months) and longer progression-free survival (6.9 months vs. 4.3 months) compared to Sorafenib in patients with unresectable hepatocellular carcinoma. Sorafenib has been the standard treatment to date. Grade 3 adverse effects, such as gastrointestinal bleeding and gastric ulcer perforation, were observed in 43% of patients treated with Atezolizumab and Bevacizumab. However, these effects were considered manageable, and clinically significant survival benefits were obtained with an acceptable safety profile. In addition, a biomarker analysis was performed that revealed a high expression of VEGF receptor 2 was associated with an increased benefit of Atezolizumab and Bevacizumab therapy. These findings support the efficacy of this combination and the importance of identifying biomarkers for patient selection.
[35]	2021	1657	This article focuses on the use of immunotherapy as a treatment for advanced HCC, with a specific focus on special subgroups of patients. It explores how immunotherapy has emerged as a promising option in the treatment of HCC and highlights the importance of considering the specific characteristics of patient subgroups, such as those with viral infections or certain comorbidities. Advances and challenges in the use of immunotherapy in these special subgroups are discussed, and the need for personalized care in the management of advanced HCC is highlighted.	This study evaluated the combination therapy of Atezolizumab and Bevacizumab in patients with HCC and found a significant increase in the overall survival of these patients. However, adverse effects were also identified, such as upper gastrointestinal bleeding, arterial hypertension, and proteinuria, which led to the suggestion that this therapy should be contraindicated in patients with HCC who have previously received an organ transplant. In addition, it was noted that the presence of elevated VEGF levels could potentiate the first-line systemic treatment of HCC and was considered a relevant prognostic factor in the response to therapy.
[36]	2022	296	The AB- real study has provided strong evidence of reproducible safety and efficacy of the combination of Atezolizumab and Bevacizumab in the treatment of HCC in clinical practice. These results support the usefulness of this therapy in a real clinical setting, highlighting its safety profile and demonstrated efficacy in HCC patients.	The median duration of treatment was 7.3 months, while the median overall survival reached 15.7 months. It was reported that 74.6% of patients experienced therapy-related adverse effects, among which bleeding was reported in 8.4%, proteinuria in 30.4%, and hypertension in 28.3%. A significant finding of the study was that those patients who achieved a radiologically appreciable response experienced greater survival. These results suggest that the combination of Atezolizumab and Bevacizumab may be effective in patients with HCC of viral etiology, with improved survival in those who respond positively to treatment.
[37]	2022	779	This international study evaluated the efficacy of the combination of Atezolizumab and Bevacizumab compared to Lenvatinib or Sorafenib in the treatment of unresectable nonviral HCC. A propensity score-matched method was used to match groups of patients with similar characteristics. The results of this analysis suggest that Atezolizumab plus Bevacizumab therapy may be a promising option in the treatment of unresectable nonviral HCC, providing valuable information for clinical decision-making in patients with this disease.	The study found that patients treated with Lenvatinib showed a superior overall survival rate compared to those treated with Atezolizumab and Bevacizumab. However, no statistically significant differences were observed between the Atezolizumab- and Bevacizumab-treated group and the Sorafenib-treated group in terms of overall survival. These findings suggest that Lenvatinib may be more effective than the combination of Atezolizumab and Bevacizumab in the treatment of advanced HCC of nonviral etiology, while the efficacy of Atezolizumab and Bevacizumab is like that of Sorafenib in this context.
[38]	2023	1334	In this meta-analysis, immunological combinations were evaluated in comparison with Sorafenib as the first-line treatment for patients with advanced hepatocellular carcinoma. The results of the analysis suggest that immunologic combinations may be a promising option in terms of efficacy for the initial treatment of this disease. This study provides valuable information on the alternative therapies available for advanced hepatocellular carcinoma and may be relevant for clinical decision-making in this setting.	Alternative treatments (*n* = 1334) were found to reduce the risk of death by 27% (HR, 0.73; 95% CI, 0.65–0.83; *p* < 0.001), in addition to increasing both the overall survival and complete response rate compared to Sorafenib (HR, 0.64; 95% CI, 0.5–0.84; *p* < 0.001) and (12.4; 95% CI, 3.02–50.85; *p* < 0.001) respectively. These findings suggest that immune combinations may be more effective in terms of survival and complete response in patients with advanced hepatocellular carcinoma compared to Sorafenib.

AFP: alpha-fetoprotein, CI: confidence interval, HCC: hepatocellular carcinoma, HR: hazard ratio, PD: programmed death, PDL1: programmed death-ligand 1, VEGF: vascular endothelial growth factor, TGF: transformation growth factor, TGF-β: transformation growth factor β, *n*: patients.

**Table 2:** Studies appraised using the Joanna Briggs Institute critical appraisal checklist for randomized controlled trials.

Study	Was True Randomization Used for Assignment of Participants to Treatment Groups?	Was Allocation to Treatment Groups Concealed?	Were Treatment Groups Similar at the Baseline?	Were Participants Blind to Treatment Assignment?	Were Those Delivering Treatment Blind to Treatment Assignment?	Were Outcomes Assessors Blind to Treatment Assignment?	Were Treatment Groups Treated Identically Other Than the Intervention of Interest?	Was Follow-up Complete, and If Not, Were Differences between Groups in Terms of Their Follow-up Adequately Described and Analyzed?	Were Participants Analyzed in the Groups to Which They Were Randomized?	Were Outcomes Measured in the Same Way for Treatment Groups?	Were Outcomes Measured in a Reliable Way?	Was Appropriate Statistical Analysis Used?	Was the Trial Design Appropriate, and Any Deviations from the Standard RCT Design (Individual Randomization, Parallel Groups) Accounted for in the Conduct and Analysis of the Trial?	Score out of 13 (100%)
Finn et al., 2020 [27]	Y	Y	Y	Y	Y	Y	Y	Y	Y	Y	Y	Y	Y	100%
Piñero et al., 2020 [28]	Y	Y	Y	Y	Y	Y	Y	Y	Y	Y	Y	Y	Y	100%
Lee et al., 2020 [29]	Y	Y	Y	Y	Y	Y	Y	Y	Y	Y	Y	Y	Y	100%
Sonbol et al., 2020 [30]	Y	Y	Y	Y	Y	Y	Y	Y	Y	Y	Y	Y	Y	100%
Da Fonseca et al., 2021 [31]	Y	Y	Y	Y	Y	Y	Y	Y	Y	Y	Y	Y	Y	100%
Han et al., 2021 [32]	Y	Y	Y	Y	Y	Y	Y	Y	Y	Y	Y	Y	Y	100%
Fulgenzi et al., 2021 [33]	Y	Y	Y	Y	Y	Y	Y	Y	Y	Y	Y	Y	Y	100%
Cheng et al., 2022 [34]	Y	Y	Y	Y	Y	Y	Y	Y	Y	Y	Y	Y	Y	100%
Pinter et al., 2021 [35]	Y	Y	Y	Y	Y	Y	Y	Y	Y	Y	Y	Y	Y	100%
Fulgenzi et al., 2022 [36]	Y	Y	Y	Y	Y	Y	Y	Y	Y	Y	Y	Y	Y	100%
Rimini et al., 2022 [37]	Y	Y	Y	Y	Y	Y	Y	Y	Y	Y	Y	Y	Y	100%
Rizzo et al., 2023 [38]	Y	Y	Y	Y	Y	Y	Y	Y	Y	Y	Y	Y	Y	100%

Y = Yes. N = No. U = Unclear.

**Table 3:** Hazard ratio for the overall survival of the analyzed studies.

Studio	Monotherapy Group	Hazard Ratio for Overall Survival	*p*-Value
Finn et al., 2020 [27]	Sorafenib	0.59; 95% CI, 0.47 to 0.76	<0.001
Piñero et al., 2020 [28]	Sorafenib	0.58; 95% CI, 0.42 to 0.79	<0.001
Lee et al., 2020 [29]	Atezolizumab	0.55; 80% CI, 0.40 to 0.74	0.011
Sonbol et al., 2020 [30]	Nivolimab Lenvatinib Sorafenib Linifanib Sunitinib	0.68; 95% CI, 0.48 to 0.98 0.63; 95% CI, 0.44 to 0.89 0.58; 95% CI, 0.42 to 0.80 0.55; 95% CI, 0.39 to 0.78 0.45; 95% CI, 0.32 to 0.63	NR
Han et al., 2021 [32]	Lenvatinib	0.63; 95% CI, 0.44 to 0.89	NR
Fulgenzi et al., 2021 [33]	Sorafenib	0.59; 95% CI, 0.40 to 0.76	<0.001
Cheng et al., 2022 [34]	Sorafenib	0.58; 95% CI, 0.42 to 0.79	<0.001
Pinter et al., 2021 [35]	Sorafenib	0.95; 95% CI, 0.74 to 1.22	<0.001
Rimini et al., 2022 [37]	Lenvatinib	0.65; 95% CI, 0.44 to 0.95	0.0268
Rizzo et al., 2023 [38]	Sorafenib	0.73; 95% CI, 0.65 to 0.83	<0.001

NR: not reported.

## Data Availability

Not applicable.
